# Imaginary Diseases

**Published:** 1881-03

**Authors:** 


### IMAGINARY DISEASES.

I BELIEVE that two persons in three who consider themselves invalids have no serious ailments, and that their diseases exist to a great extent in the imagination only.

Hardly a day passes that I do not see this opinion verified. There is often some disturbance of the system with those who ask advice; but they are not usually afflicted in the manner they had supposed, and most frequently not seriously ill in any respect except as to the imagination.

I have never suffered an hour from any sort of illness, since acquiring the knowledge and experience which enable me to account for many of the seeming phenomena incident to human life ; previous to that the monotony of my life would be occasionally interrupted by a scare, from which I would suffer during the time it required to reach the nearest competent physician. My imagination has thus presented me at various times with heart disease, kidney troubles and liver complaint, each of which I left behind me when I came away from the doctor's, and have never heard from since.

Few people have any idea of the aggregate of suffering and misery that is silently endured by thousands of our fellow beings, under the supposition that they are victims of incurable diseases, when in fact they have nothing serious the matter with them.

Every physician of experience is able to refer to many cases where he has been able to lift a terrible weight from the crushed spirit of some suffering mortal, by explaining away his fears. It should be more satisfactory to a humane physician to quiet the fears of one imaginary invalid, than to have a hundred *rich* patients who required his services.

The JOURNAL OF HEALTH was founded with the beneficent object of teaching people how to avoid disease when avoidable, and when disease comes, it has always advised that some competent physician should be promptly consulted. If its suggestions were always heeded there would be but few cases of incurable diseases among its readers. I venture to say that seventy cases of disease in every hundred are preventable, and that ninety-five in every hundred are curable by prompt and judicious treatment.

As to imaginary diseases, let it not be thought that I would advise any person to quietly convince himself that there was nothing the matter, and thus silence his fears ; there might be some serious trouble, and then the advantage of early treatment would be lost. A skillful physician should be consulted in every doubtful case. The probability is he would find the patient suffering in most cases from dyspepsia instead of heart disease, or from malaria instead of Bright's disease of the kidneys ; that is, from a curable instead of an incurable disease.

